# Rule-Based Models for Risk Estimation and Analysis of In-hospital Mortality in Emergency and Critical Care

**DOI:** 10.3389/fmed.2021.785711

**Published:** 2021-11-08

**Authors:** Oliver Haas, Andreas Maier, Eva Rothgang

**Affiliations:** ^1^Department of Industrial Engineering and Health, Institute of Medical Engineering, Technical University Amberg-Weiden, Weiden, Germany; ^2^Pattern Recognition Lab, Department of Computer Science, Technical Faculty, Friedrich-Alexander University, Erlangen, Germany

**Keywords:** in-hospital mortality, critical care, odds ratio, associative classification, machine learning, artificial intelligence

## Abstract

We propose a novel method that uses associative classification and odds ratios to predict in-hospital mortality in emergency and critical care. Manual mortality risk scores have previously been used to assess the care needed for each patient and their need for palliative measures. Automated approaches allow providers to get a quick and objective estimation based on electronic health records. We use association rule mining to find relevant patterns in the dataset. The odds ratio is used instead of classical association rule mining metrics as a quality measure to analyze association instead of frequency. The resulting measures are used to estimate the in-hospital mortality risk. We compare two prediction models: one minimal model with socio-demographic factors that are available at the time of admission and can be provided by the patients themselves, namely gender, ethnicity, type of insurance, language, and marital status, and a full model that additionally includes clinical information like diagnoses, medication, and procedures. The method was tested and validated on MIMIC-IV, a publicly available clinical dataset. The minimal prediction model achieved an area under the receiver operating characteristic curve value of 0.69, while the full prediction model achieved a value of 0.98. The models serve different purposes. The minimal model can be used as a first risk assessment based on patient-reported information. The full model expands on this and provides an updated risk assessment each time a new variable occurs in the clinical case. In addition, the rules in the models allow us to analyze the dataset based on data-backed rules. We provide several examples of interesting rules, including rules that hint at errors in the underlying data, rules that correspond to existing epidemiological research, and rules that were previously unknown and can serve as starting points for future studies.

## 1. Introduction

The term in-hospital mortality defines the death of a patient during their stay at the hospital. Especially in emergency and critical care, many patients die in the hospital. While not all of these deaths can be prevented, early knowledge of a patient's in-hospital mortality risk can be used to assess the patient's status and necessary adjustments to this patient's care, reducing missed care and decreasing mortality rates (1). Apart from individual changes like the start of palliative care, organizational changes like a different allocation of nurse time or other resources can be informed by such risk scores. In-hospital mortality rates have also been used in the assessment of hospital care quality, as is the case in the United Kingdom (2).

In-hospital mortality risks are commonly determined manually (3). Manual scoring systems are built upon expert knowledge and have gone through significant development time. Another approach is Machine Learning (ML), which uses data and statistical methods to build a predictive model (4). Several methods for ML-based methods have been developed in recent years (5–7). While these approaches offer data-based evidence that is independent of expert knowledge, they face two challenges.

First, they often lack interpretability. As critical care is a life-and-death situation, providers need to be able to understand why a patient's status is assessed the way it is. Interpretability in Machine Learning is a broad field. Two distinctions to be made are local vs. global interpretability, i.e., whether the interpretation concerns one observation or the whole population, and model-specific vs. model-agnostic interpretability, i.e., whether the interpretation comes from within a specific model or is built on top of an existing model (8). We consider global model-specific interpretability. This offers two decisive advantages. First, global interpretability allows us to not only make predictions based on data, but also to explore the complex and heterogeneous data underlying our prediction model. Second, model-specific interpretability allows us to explain the reasoning behind our model and its inner workings to providers. Interpretability in in-hospital mortality risk estimation has previously been discussed (6, 9) and a trade-off between a prediction model's interpretability and predictive performance has been identified. Among the commonly used algorithms, Decision Trees (10) often offer high predictive performance while being highly interpretable (6).

The second challenge arises from the high number of possible variables in in-hospital mortality risk estimation. As critical care is complex, many variables can potentially play a role in estimating a patient's in-hospital mortality risk. This renders many common ML algorithms challenging to use and increases models' complexity, further hindering interpretability. Manual methods use expert knowledge from years of scientific research to identify which variables to include. Variables that were previously not considered but are readily available could play a role in in-hospital mortality risk estimation because they are highly correlated.

Association rule mining (ARM) is often used to detect patterns in high-dimensional data (11). This data mining method analyzes a given dataset for rules of the form “*A*⇒*B*,” where *A* and *B* are sets of *items*, which in our case describe variable-value pairs. Such a rule denotes that in an observation in the dataset in which the items in *A* occur, the items in *B* will also likely occur. This algorithm produces a set of rules that fulfill pre-configured quality constraints. In the neighboring field of associative classification (AC), this class of algorithms is used to mine rules that help in the classification task at hand (12). AC algorithms first mine rules in which the right-hand side is the outcome of interest and then build a classifier based on these rules, e.g., by using the best rule that applies to an observation or by aggregating all applying rules (12). This leads to predictive models that are easy to interpret and offer a human-readable, model-specific, and global interpretation. Additionally, the rules in the model can be used to analyze the dataset itself due to their statistical nature. This helps detect interesting patterns in the data that correspond to correlations between clinical variables and the outcome of interest.

AC methods have previously been used in various fields of healthcare, including the prediction of outcomes and adverse events (13–16), the prediction of diseases and wellness (17–20), as well as biochemistry and genetics (21–25). AC methods have also been used in the field of in-hospital mortality risk estimation using the results of 12 lab tests (26). This shows the feasibility of AC methods in in-hospital mortality risk estimation.

We aim to improve automated, ML-based in-hospital mortality risk estimation methods by including heterogeneous variables as well as more variables in general. ARM methods, and thus also AC models, can incorporate large numbers of variables of different types, which is why we analyze the feasibility of AC models for in-hospital mortality risk estimation. We propose to use AC models to estimate the risk for in-hospital mortality risk estimation in critical and intensive care. The goal of the present study is 1) to analyze whether this approach is feasible and 2) which kind of rules are found to by such methods and which variables play a role in the prediction. This expands our previous work (27) in major ways. First, we present two models to analyze the temporal evolution of the prediction. Second, we expand our analysis of the resulting rules. Third, we compare our models to Decision Tree models. Lastly, we add more experiments to gain more insight into the model size.

## 2. Materials and Methods

**Figure 1:**
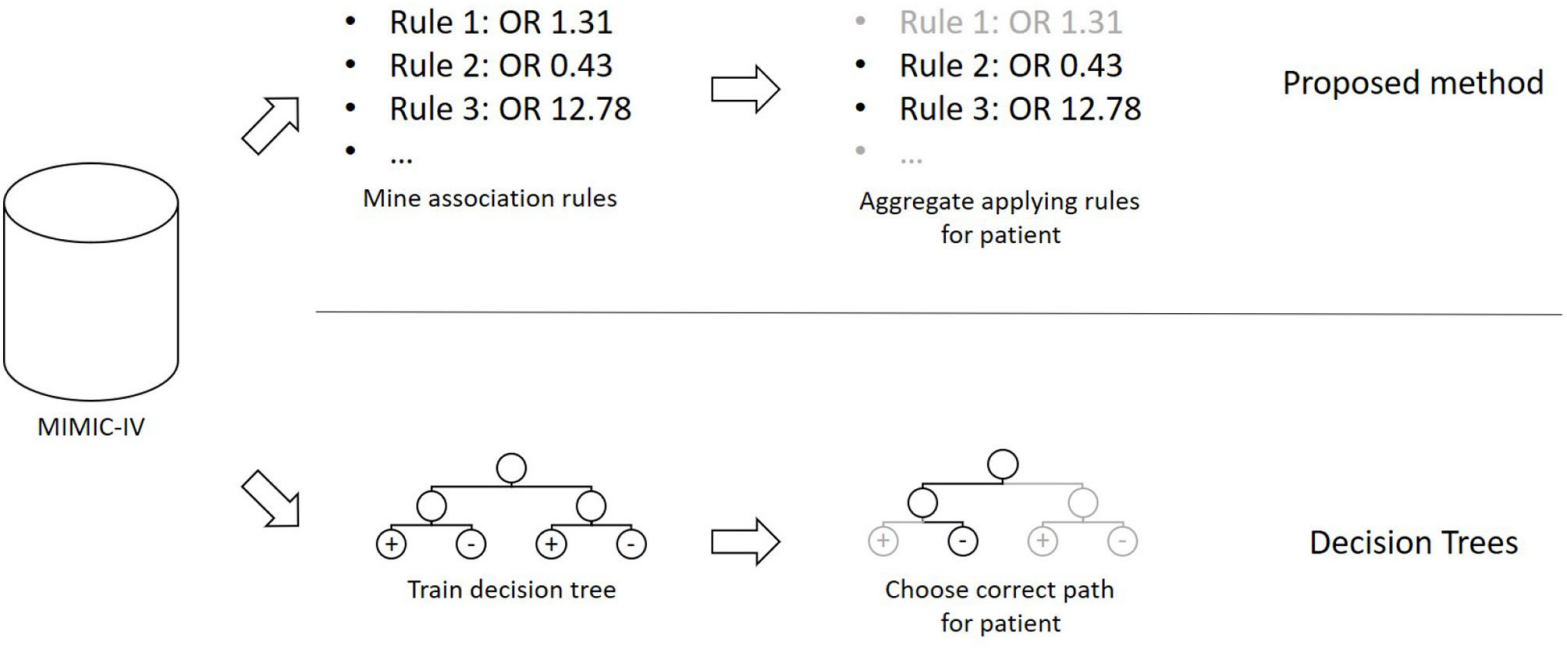
The overall concept of the method in comparison to decision trees.

The method presented in this section was developed in the programming language C#. The overall concept of the proposed method, as well as a comparison to Decision Trees, can be seen in Figure 1. Based on a publicly available clinical dataset, we first mine association rules from the data. The resulting rules are then combined into a prediction model that can be applied to previously unseen cases. Finally, we test and validate the proposed method using experiments.

### 2.1. Dataset

We used data from the MIMIC-IV project, version 0.4 (28). MIMIC-IV is a collection of around 525,000 emergency department and intensive care unit cases, collected between 2008 and 2019 at the Beth Israel Deaconess Medical Center in Boston, Massachusetts, United States of America. Recorded variables include diagnoses, procedures, drug prescriptions, socio-demographic factors like gender, insurance type, and marital status, and organizational information like diagnosis-related groups (DRGs), wards, and services.

Table 1 lists all types of variables used in this study. We included all categorical information that can be assumed to be available in most clinical contexts. This excludes rapidly changing information like vital parameters as well as textual notes. Not all of this information is available at the beginning of a clinical case. Some information, like diagnoses, is recorded later in the case. We thus divided the variable types into two classes. In the first class, only variables that are available at the beginning of the cases are considered. These will be used to build a minimal model to estimate the in-hospital mortality risk right at the beginning of the case. The second class contains all variable types and is used to build a full model based on all available information.

**Table 1:** A list of all variables from MIMIC-IV that were used in this study.

**Variable type**	**Minimal model?**	**Description**	**# variables**
Diagnosis		Diagnoses (coded as ICD)	86,751
Ethnicity	✓	White, Asian, Black/African American etc.	8
Gender	✓	Binary: male/female	2
Insurance type	✓	Medicare, Medicaid, or Other	3
Language	✓	Binary: English/other	2
Marital status	✓	Single, married, divorced, widowed, or missing	5
Prescription		Drugs described to a patient	10,259
Procedure		Procedures (coded as ICD)	82,763
Service		Clinical services: neonatal, psychological etc.	21
Ward		Clinical wards: intensive care unit, surgery etc.	43
Overall	5 of 10		179,857

*The table includes the name of the variable type, whether it is part of the minimal model, a description, as well as the number of variables. ICD International Classification of Diseases*.

All cases have been transformed into a set of items to enable ARM. This was done by adding all variables that occurred during a case to the case's itemset. We did not exclude any case in order to get a general in-hospital mortality risk estimation model.

This results in 179,857 clinical variables and 524,520 cases, in 9,369 (1.79%) of which the patient died during their stay in the hospital. This in-hospital mortality rate is comparable to similar populations like England (2).

### 2.2. Rule Mining

Due to the presence of rare diseases or smaller patient subgroups, infrequent rules can be of interest in healthcare. This is why we do not use the classical ARM metrics like support and confidence (11) that focus on frequency, but instead epidemiological metrics that are widely used to measure associations between clinical variables and outcomes. We use the odds ratio (OR) as the primary metric to measure this association. Odds ratios have previously been used in ARM in healthcare (13). Starting from a contingency table like the one in Table 2, the OR can be calculated as


(1) $$\begin{array}{l} \text{OR}=\frac{ad}{bc}=\frac{100\cdot 400}{200\cdot 300}=\frac{4}{6}=0.\bar{6}, \end{array}$$

indicating that there is a negative association between the items in *A* and in-hospital mortality. The set *A* can contain one or more items of one or more different types. This flexibility makes ARM techniques easy to use with large, heterogeneous data. ORs range from 0 to +∞. An OR of 1.0 denotes no classification, while an OR ≤ 1.0 denotes a positive or negative association, respectively. The higher (in the case OR > 1.0) or lower (OR < 1.0), the stronger the association.

**Table 2:** A fictional contingency table to show how the OR is calculated.

	**Died**	**Survived**
Items in *A* occurred	*a* = 100	*b* = 200
Items in *A* did not occur	*c* = 300	*d* = 400

The mining process consists of two steps. In the first step, a contingency table like the one in Table 2 is constructed for each variable by counting the cases with and without the variable as well as with and without in-hospital mortality. From this table, the odds ratio is calculated according to Equation (1). Note that this also includes ORs lower than 1.0, which denotes a negative association. ORs of 0 or ∞ are discarded. This corresponds to one of the cell entries *a, b, c* and *d* being 0.

In the second step, the model size is reduced by applying a filter to the rules constructed in the first step. As an OR of 1.0 denotes no association, we use a statistical hypothesis test to ensure that the calculated OR differs significantly from 1.0. The normal approximation of the log odds ratio (29) is used. The null hypothesis is “OR = 1.0,” and the test returns a two-sided *p*-value. Only ORs with a *p*-value below a configurable value *p*_max_ are kept. As this method results in many tests on the same dataset, Bonferroni correction (30) can be used. This procedure divides *p*_max_ by the number of tests to be executed and uses this quotient as the threshold value instead of *p*_max_. All rules of the form “variable⇒in-hospital mortality,” together with their OR, that are left after this filtering step then form the prediction model.

### 2.3. Prediction

Given a new observation, the prediction model can be used to estimate the corresponding patient's in-hospital mortality risk using the model's rules. First, all the rules that apply to the model are determined. The remaining rules do not play a role in this observation's prediction. The ORs of these applying rules are then aggregated by calculating their average value. A decision boundary δ is used to decide which average OR leads to the prediction of high in-hospital mortality risk. If OR_*p*_ is the average OR of all rules that apply to observation *p*, then the prediction model's decision function *f*(*p*) is


(2) $$f(p)=\{\begin{array}{ll} \text{high in-hospital mortality risk,} & {\text{if OR}}_{p}\geq \delta ,\\ \text{low in-hospital mortality risk,} & {\text{if OR}}_{p}<\delta . \end{array}$$

### 2.4. Experiments

The following hyperparameters were used. The *p*-value threshold *p*_max_ decides which rules are kept in the filtering step. We used the thresholds 10^−*n*^ for *n* in {0, 1, …, 10} as well as the commonly used value 0.05. All of these thresholds were used with and without Bonferroni correction. Additionally, a value of 0 was used to only keep ORs with a *p*-value of exactly 0. This was possible due to the high number of observations. As computers use finite representations of floating-point numbers, this should be understood to be exactly zero after internal rounding. This resulted in 13·2 = 26 experiments. Each experiment was repeated ten times. Each time, the dataset was randomly split into 90% training set and 10% test set. The primary metric of interest was the area under the receiver operating characteristic curve (AUC) (31). The AUC values were calculated using the Accord Framework, version 3.8.0 (32). All metrics have been calculated both on the training and the test set to analyze possible overfitting. The experiments were executed for both the full model and the minimal model.

## 3. Results

**Figure 2:**
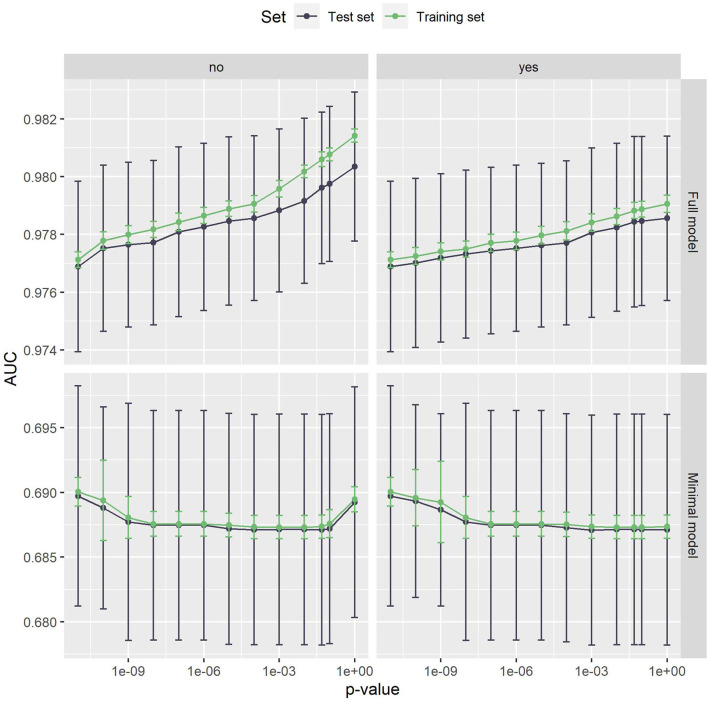
The AUC values of the full model (top) and the minimal model (bottom). Error bars show one standard deviation, the color indicates whether the test or the training set was used. The left panel is without, the right panel with Bonferroni correction. The x-axis shows different *p*-values used in the filtering step. The leftmost dot denotes the *p*-value zero. The full model clearly outperforms the minimal model. Both models are very stable with respect to both the *p*-value threshold and Bonferroni correction.

The AUC values achieved by the proposed method can be seen in Figure 2. For the full model, the mean AUC values on the test set range from 0.977 to 0.980, which shows that the filtering step has a minor impact on the predictive performance. With a range of 0.687 to 0.690, this is also true for the minimal model. In both cases, the difference to the training set is low, indicating that the method does not suffer from overfitting. The largest deviation in mean AUC values was 0.001 for the full model, *p*_max_ = 1 and no Bonferroni correction.

**Figure 3:**
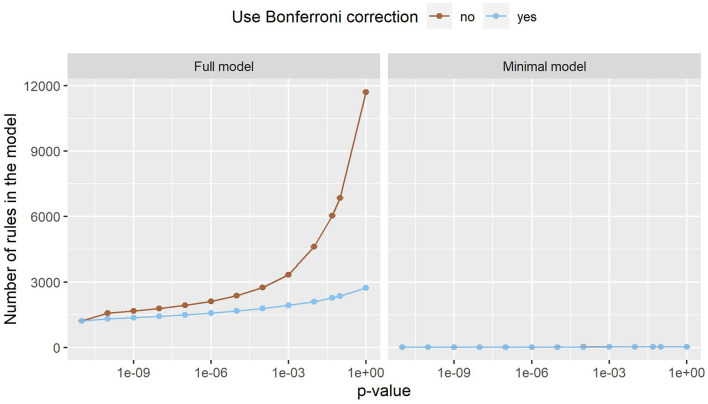
The number of rules the full model (left) and the minimal model (right). Error bars (which are very small) show one standard deviation, the color indicates whether Bonferroni correction was used. The x-axis shows different *p*-values used in the filtering step. The leftmost dot denotes the *p*-value zero. The number of rules increases exponentially in the full model, while this effect is much smaller in the minimal model. The minimal model has fewer rules than the full model.

**Figure 4:**
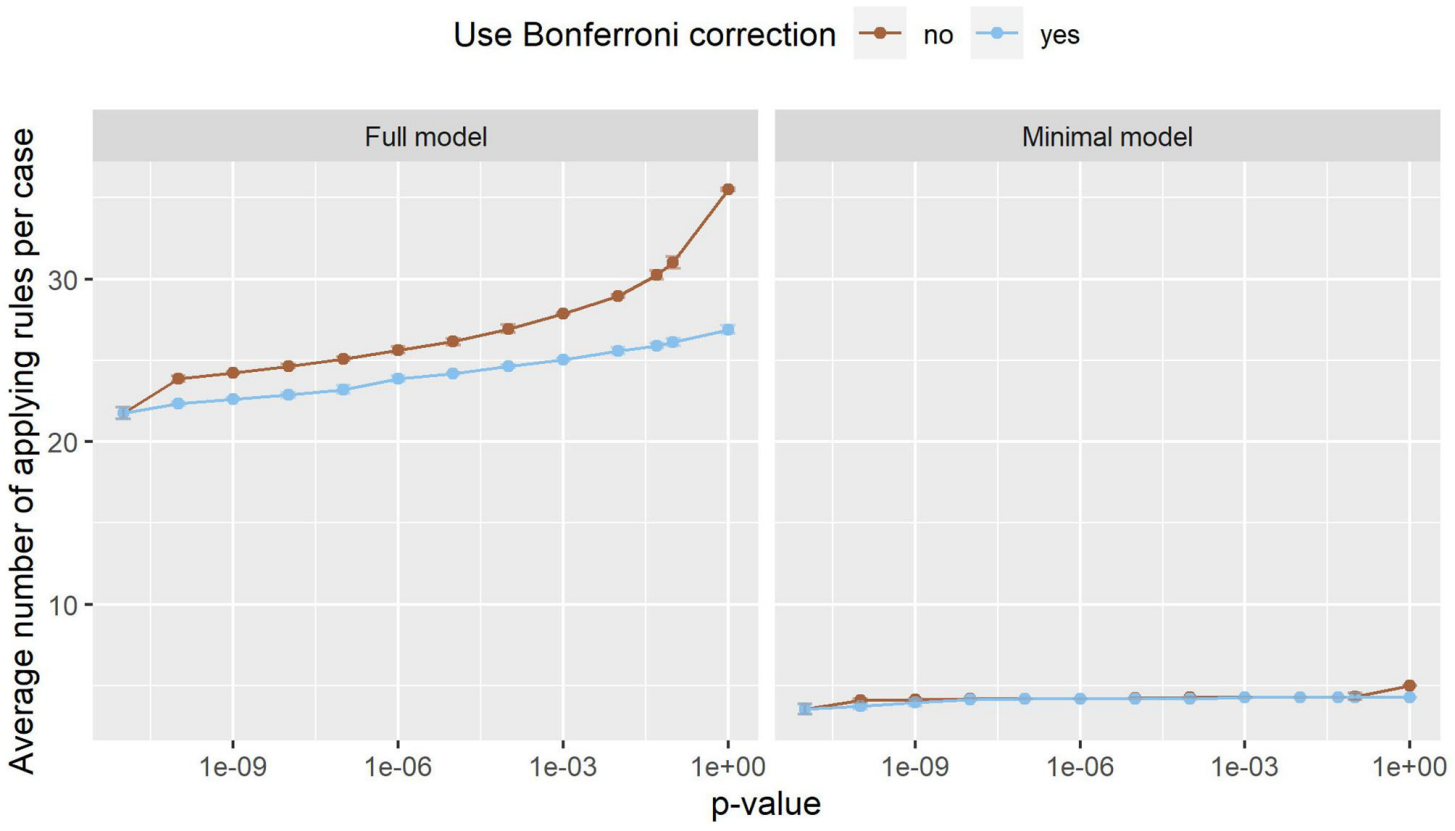
The number of rules that apply to an average case in the full model (left) and the minimal model (right). Error bars (which are very small) show one standard deviation, the color indicates whether Bonferroni correction was used. The x-axis shows different *p*-values used in the filtering step. The leftmost dot denotes the *p*-value zero. Only a small fraction of all rules apply to an individual case, making it easy to interpret the model and its decisions.

Figure 3 shows the number of rules in both models. In the minimal model, the number of rules is almost constant. This can be explained by the small number of variables, as only 20 variables are considered. In the full model, on the other hand, almost 180,000 variables can be included in the model. The number of rules grows exponentially, with Bonferroni correction slowing the growth down considerably. Still, the number of rules reaches into the thousands. As such a large number of rules is hard to handle, a strict *p*-value filter is advisable if interpretability is of interest. As the performance stays almost the same with lower *p*_max_ values, but the number of rules is considerably lower, we can deduce that the statistical significance test is an effective filter that greatly reduces the size of the prediction model without compromising the predictive performance. Still, around 1,220 rules remain even for the full model with *p*_max_ = 0. As can be seen in Figure 4, this is due to the complexity of the problem. In-hospital mortality can be caused and influenced by many factors. Per observation, however, only around 22 to 36 rules apply when using the full model, depending on *p*_max_ and the use of Bonferroni correction. On average, around 4 to 5 rules apply when using minimal model.

In the remainder of this study, we further analyze small prediction models, as they are easier to manage and interpret and have a comparable predictive performance. For both the full model and the minimal model, the lowest threshold *p*_max_ = 0 was used. In this case, Bonferroni correction makes no difference. This results in a full model with 1,217 rules and an AUC of 0.98 and a minimal model with 13 rules and an AUC of 0.69.

**Figure 5:**
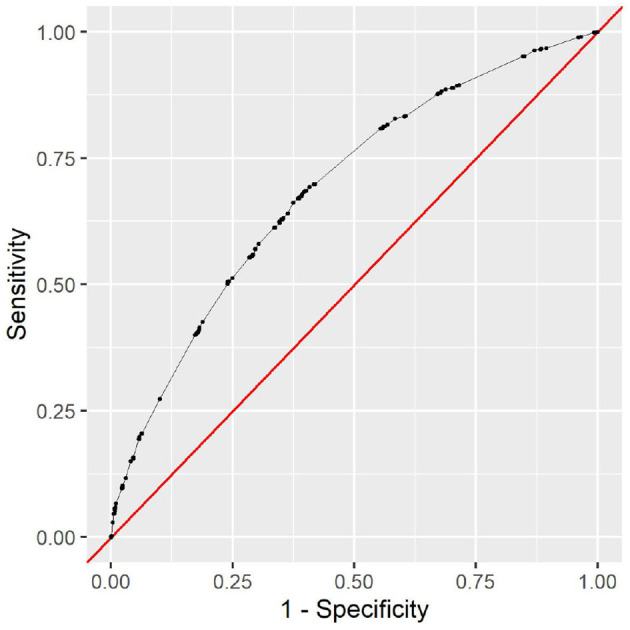
The receiver operating characteristic of the minimal model.

The full model clearly outperforms the minimal model. This is to be expected, as the minimal model only contains very limited data. The variable types ethnicity, gender, insurance type, language, and marital status contain no information that could help determine the cause of the clinical stay or the patient's health status. However, it is noteworthy that the minimal model still achieves an AUC of 0.69 with this limited information. As all this information is readily available and patient-reported, the model can be used as a first assessment before any provider encounter. The corresponding receiver operating characteristic (ROC) curve can be seen in Figure 5. To choose a decision boundary, we searched for the highest Youden index (33), which is equivalent to optimizing the sum of sensitivity and specificity. The corresponding minimal model uses a decision boundary of 1.015, which results in an accuracy of 63%, a sensitivity of 66%, and a specificity of 63%. Other decision boundaries are possible, depending on the context in which they are used. Varying the decision boundary will affect both sensitivity and specificity of the model.

**Figure 6:**
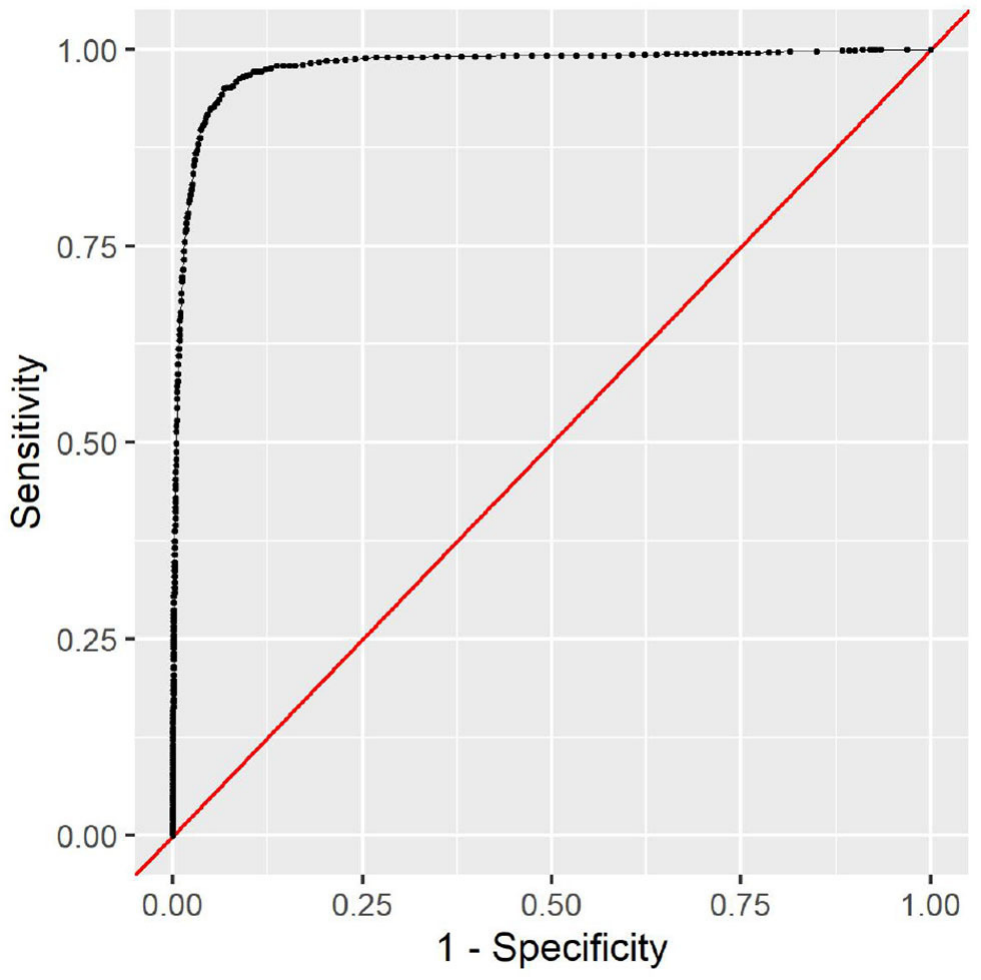
The receiver operating characteristic of the full model.

This additional information in the full model improves the predictive performance considerably. With an AUC of 0.98, the model can almost perfectly predict the in-hospital mortality risk. The corresponding ROC curve can be seen in Figure 6. With an optimal decision boundary of 5.306, this results in an accuracy of 93%, a sensitivity of 95%, and a specificity of 93%.

All analyses were done in the R programming language (34), using the tidyverse packages (35). The rules of both models can be found in the Supplementary Materials.

### 3.1. Analysis of Rules

Apart from their predictive qualities, the rules in the models also allow us to analyze the algorithm's reasoning. The 13 rules in the minimal model span all five included variable types. These rules indicate that male patients die more often than female patients (ORs 1.25 vs. 0.80), that English speakers are more likely to survive than others (0.73 vs. 1.36), and that Medicare patients are more likely to die than Medicaid and other patients (2.40 vs. 0.57 vs. 0.50). The ethnicities ‘Hispanic/Latino’ and ‘Black/African American’ are negatively associated with in-hospital mortality (0.47 and 0.58), while ‘unknown’ and ‘unable to obtain’ have higher ORs (4.59 and 2.25). The marital status ‘widowed’ is positively associated with in-hospital mortality (2.05), while being single is negatively associated with in-hospital mortality (0.50).

From the five variable types in the minimal model, the same 13 rules are in the full model. The remaining 1,204 rules span over the five other variable types with 602 diagnosis, 445 prescription, 131 procedure, nine service, and 17 ward rules.

Only five diagnosis rules are negative associations, namely three diagnoses on single liveborn infants (0.08-0.16), encounters for immunizations (0.06), and unspecified chest pain (0.11). The remaining 597 rules can be categorized into various groups of rules that describe variants of the same pattern. Examples include alcohol abuse and its consequences (2.34–11.69), anemia (1.67–4.56), various forms of hemorrhage (3.32–582.13), neoplasms (3.50–13.66), pneumonia (6.27–18.49), pressure ulcers (5.29–17.96), sepsis and septicemia (4.15–33.18), and diabetes (1.56–13.20). Some doubling occurs due to two versions of the International Classification of Diseases being used in the dataset.

This also concerns the 131 procedure rules. Here, examples of groups of rules are catheterizations (2.59–21.11), drainages (5.53–22.06), ventilation (2.44–26.02), and transfusions (3.66–13.51). No procedure rule has an OR below 1.0.

Only 17 out of 445 prescription rules are negative. As some drugs are recorded with slightly different names, different doses or different mode of administration, some doubling occurs. One extreme example is sodium chloride with 20 rules. The rules “0.45% Sodium Chloride” and “0.45 % Sodium Chloride” (note the space) have ORs of 2.11 and 94.94, respectively. The difference in ORs can be explained by the unequal distribution across units. While 18 of the 23 (78%) cases with “0.45 % Sodium Chloride” in MIMIC-IV were in at least one Intensive Care Unit, only 3375 of the 15,180 (22%) cases with “0.45% Sodium Chloride” were in at least one Intensive Care Unit. This indicates that there are some unexpected inconsistencies in the data. With almost 180,000 variables, it would be impossible to check all variables manually for inconsistencies due to variations in practice. Other prescription rule groups with high variation include Heparin (2.10–68.58), vaccines (0.01–2.82), and lidocaine (2.10–15.55).

Three service rules are negative, namely obstetrics (0.01), newborn (0.17), and orthopedic (0.19). The positive rules include four medical services (1.64–2.62), trauma (2.39), and neurologic surgical (2.73).

Finally, five out of 17 ward rules are negative, with three being at least partially about childbirth (0.02–0.48). The six rules with the highest ORs (7.55–13.25) are all the intensive care units in the dataset. Both the service and ward rules reflect different patient populations with different reasons for the clinical stay. The in-hospital mortality risk is vastly different between pregnant women and traffic accident victims that need intensive care, for example.

Overall, only 37 of 1,217 rules are negative. The five rules with the lowest ORs are about childbirth (0.00–0.04) and a Hepatitis B vaccine (0.01), the five highest ORs are medications used in palliative care (Morphine and Angiotensin II, 856.26 and 332.77), subdural hemorrhage (582.14), and two rules on brain death (413.38 and 668.41). These last two rules are unexpected. Brain death should always co-occur with in-hospital mortality, which would exclude this OR of ∞. Their occurrence hints at an error in the data. Braindead patients who are organ donors are recorded as having died, but then another case is opened for them with the diagnosis of brain death, but it is recorded that they survived this second case. This leads to more inconsistent data contained in MIMIC-IV, which can now be seen in the prediction model.

### 3.2. Interpretability

To study the proposed method's interpretability, we give a fictional example of how the prediction model can be used. Table 3 shows the rules that apply to a fictional patient in the emergency department. This list of rules is how the prediction model's decision could be shown to providers. Apart from the decision, the rules also explain what the case is about. The items in the minimal model give us a first assessment. The patient is female, Black/African American, single, insured under Medicaid, and speaks English. This results in an average OR of 0.64, which is below the decision boundary of 1.015. This tells us that, based on the minimal model, our patient is low-risk.

**Table 3:** A list of rules that apply to a fictional patient.

**Variable type**	**Description**	**OR**	**Minimal model?**
Ethnicity	Black/African American	0.58	✓
Gender	Female	0.80	✓
Insurance type	Medicaid	0.57	✓
Language	English	0.73	✓
Marital status	Single	0.50	✓
Diagnosis	Acidosis	10.96	
Diagnosis	Anuria and oliguria	17.27	
Prescription	Sodium Bicarbonate	11.54	
Prescription	Furosemide	5.00	

*This is also how the applying rules could be shown to providers*.

In the full model, more information becomes available. For example, suppose that the following information has become available after 20 min in the emergency department. We now know that acidosis and anuria or oliguria was diagnosed. The patient was given Sodium Bicarbonate and Furosemide and is currently in the emergency department. While this is not much information due to the short length of stay, it allows us to update our risk estimation. With an average OR of 5.328, which is greater than the full model's decision boundary of 5.306, we conclude that the patient currently has a high in-hospital mortality risk, but she is close to the decision boundary. We see that not only are the models interpretable, but they can also be used to get a quick overview of the case at hand.

### 3.3. Comparison to Decision Trees

We compared our models to Decision Trees models, which are the state of the art in interpretable machine learning for in-hospital mortality as they show the best trade-off between performance and interpretability (6).

We trained Decision Tree classifiers using scikit-learn, version 0.24.2 (36), with varying maximal depths. As the dataset is highly imbalanced, with over 98% of patients surviving, we used the balanced class weight option to give more weight to the rarer class. These experiments were executed for both the full and the minimal model.

**Figure 7:**
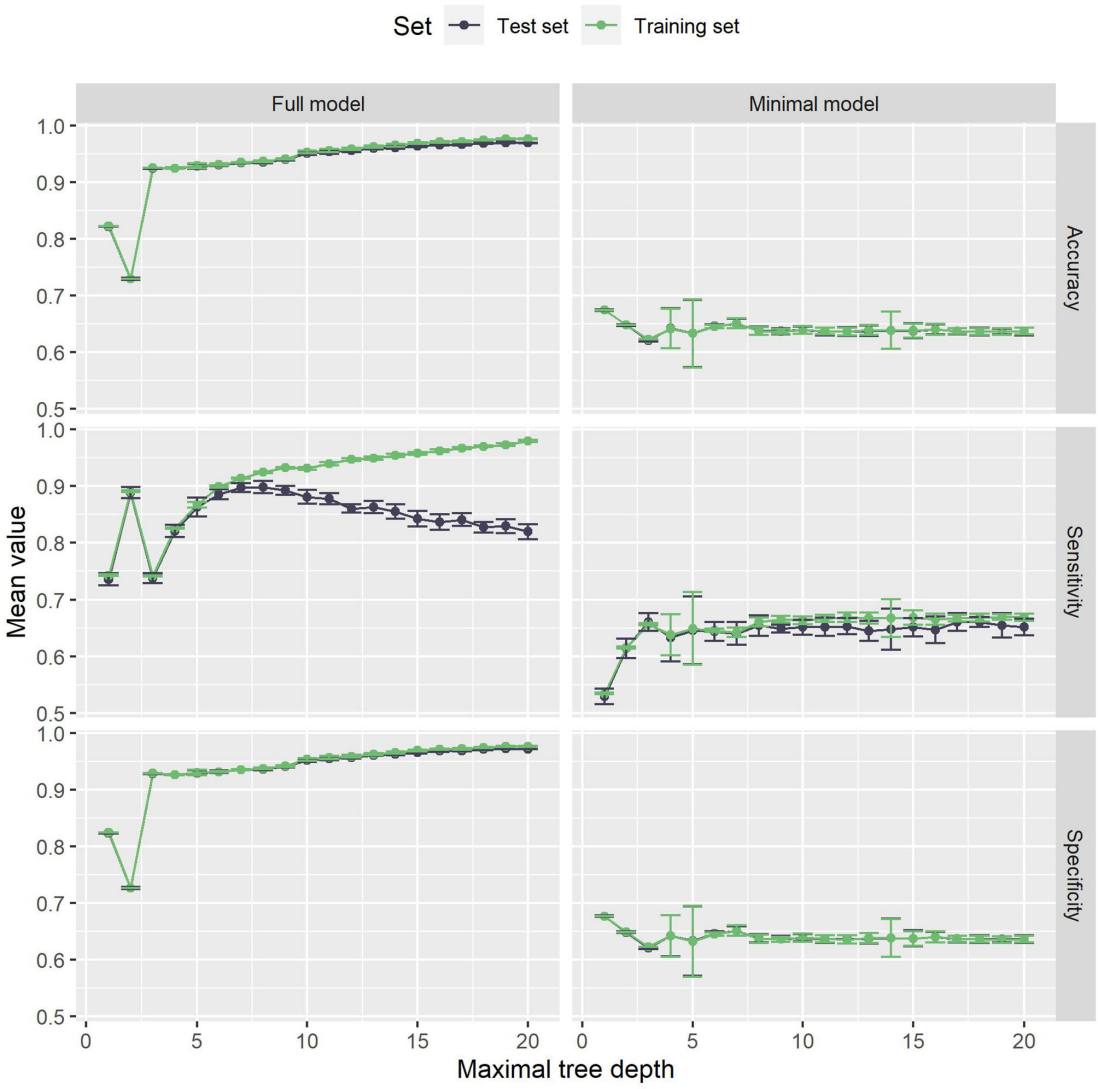
Various metrics on the Decision Tree models. The left panels are the full model, while the right panels are the minimal model. The color denotes whether the training or the test set was used to produce these metrics. On the x-axis, various maximal tree depths were analyzed. For the full model, there is major overfitting. The test set sensitivity quickly drop below the training set sensitivity for higher maximal tree depths. For the minimal model, the sensitivity also drops below the training set's sensitivity.

**Figure 8:**
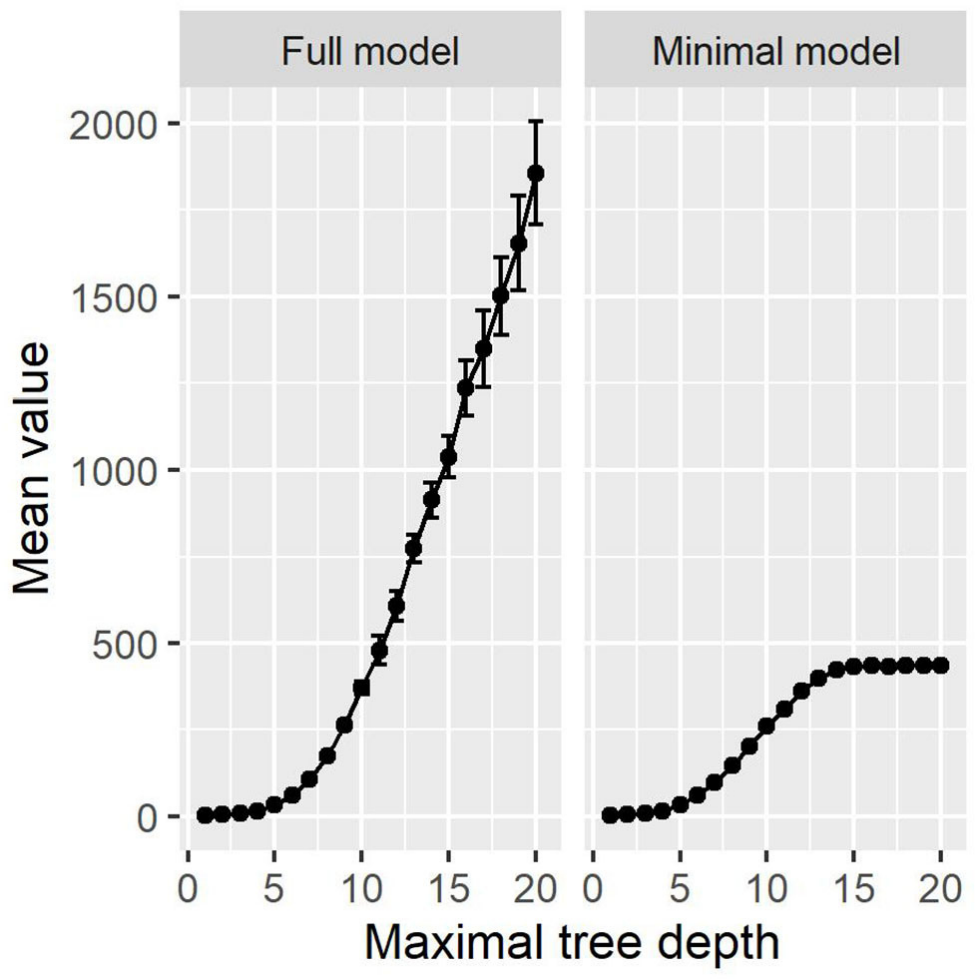
The number of leaves in the Decision Tree models. The full model increases exponentially. The minimal model's size is constant above a depth of around 15.

The results are visualized in Figure 7. For the minimal model, the results are very similar to our proposed method. Above a maximal tree depth of five, the metrics are stable, with only small deviations in the sensitivities of the training and test set. Figure 8 shows the number of leaves in the trees, which can be understood as the number of possible paths in the tree and thus as the number of rules that can be extracted from such a tree. The number of leaves in the minimal Decision Tree model grows quickly to around 433. The growth stops at a maximal tree depth of around 15, and the model's size stays relatively stable. A maximal depth of at least six is needed to achieve a stable model with low standard deviations and satisfactory metrics, at which point the model already contains around 60 leaves. Our proposed method's minimal model only needs 13 rules for comparable performance. We thus see that while the predictive performance is similar to our proposed minimal model, the complexity of the model is higher.

The full Decision Tree model suffers from major overfitting, as can be seen in the sensitivity values of the test set, which drop below the training set sensitivity for maximal tree depth values above five. With the highest sensitivity of 90%, Decision Trees cannot outperform our proposed method's full model in terms of detecting high-risk cases. On the other hand, decision Trees achieve a slightly higher specificity of 97% and thus detect low-risk cases more reliably. Note that the choice of a decision boundary impacts both sensitivity and specificity of the proposed method, and another decision boundary for the full model (namely 6.081) leads to almost the same metrics as the full Decision Tree model (namely a sensitivity of 90% and a specificity of 96%). In this way, the higher specificity at the cost of a lower sensitivity can also be achieved with the full model.

As can be seen in Figure 8, the number of leaves grows exponentially in the full model, just as the number of rules does. In comparison to Figure 3, there are more rules in our proposed method then leaves in the Decision Tree methods. With Decision Trees, however, the paths are much more complex, as their length can go up to the maximal tree depth. We provide one example full Decision Tree model with a maximal tree depth of seven in the Supplementary Materials. At this depth, the Decision Tree model does not yet suffer from major overfitting, resulting in a test accuracy of 93%, sensitivity of 90%, and specificity of 94%. The tree contains 103 leaves and 204 decision nodes.

Following the first chain in the tree, the model decides that the patient is low-risk due to the variables “5% Dextrose,” “Morphine Sulfate,” “Encounter for palliative care,” “Insertion of endotracheal tube,” “Encounter for palliative care,” and “Vasopressin” being absent in the case. If the diagnosis “Less than 24 completed weeks of gestation” were present, the model would decide that the patient is high-risk. This means that the variables' influence is combined into a chain of yes-no choices, and one change in the decision chain can alter the model's decision. This hides each variable's influence on the decision as it could occur multiple times in the tree, but each patient only activates one chain. Our proposed method separates all the variables into one-to-one rules, making it easier to identify each variable's importance individually. The combination of the variables into one decision takes place during the prediction, where the whole context of the case is taken into account and we get an overview of the case.

Another challenge in the interpretation of decision tree models is the combination of variables in each chain. The variable “Encounter for palliative care” occurs twice in the same chain due to two International Classification of Diseases versions being used. Additionally, the variable “Less than 24 completed weeks of gestation” does not match the palliative care diagnoses. The tree structure does not allow us to get an overview of the case at hand as it also highlights variables that did not occur during the case, obfuscating what actually happened. Our full model shows which rules apply to the case and thus give us an explanation of what happened during the case. Building the decision on top of this knowledge helps providers understand the model's decision.

In summary, Decision Tree models do not offer performance improvements and show limited interpretability compared to our proposed model. This is due to the fact that more complexity in the Decision Tree Models is needed to achieve a comparable predictive performance and because Decision Trees combine various variables in chains of yes-no-choices. Additionally, Decision Tree models introduce order to the variables, as the tree-like structure divides the dataset sequentially. On the other hand, our model treats every variable individually, independent of the other variables, making it easier to analyze each variable's effect.

## 4. Discussion

Associative classification based on ORs is a feasible method for in-hospital mortality risk estimation. Apart from the high predictive quality of the full model, the proposed method also allowed us to analyze the dataset based on the rules contained in the prediction model. The proposed method is not prone to overfitting and generalizes well.

While the AUCs of the minimal model are much lower, its predictive qualities are relatively high. Only socio-demographic information that can be provided by the patient is added, there is no information on the case at hand. This hints at underlying structural differences. The information what its effects are on individual patients can be valuable information for providers.

The fictional patient described above is an example of how the prediction models can be easily explained to providers. This interpretability also helps us understand the clinical data and discover patterns and inconsistencies in the data. As shown in the examples of brain death and sodium chloride, inconsistencies in the data show up as unexpected rules or rules with similar variables but vastly different ORs.

Other rules can serve as starting points for future studies. One example are the language rules. The evidence in support of an effect of primary language on in-hospital mortality is limited (37, 38). Nevertheless, our rules suggest some underlying effect exists with ORs of 0.73 for English speakers and 1.36 for others. Further research is needed to analyze the differences in these groups and whether this effect is due to documentation practice.

The majority of rules reproduce known associations or associations that are evident and explainable with expert knowledge. Examples for the latter include the higher mortality in intensive care units due to the higher number of critical cases, the low mortality in childbirth rules, the high mortality in Medicare patients due to confounding by age, and various diagnoses that are either indicative of palliative care (like Morphine or Angiotensin II) or of low-risk cases like immunizations.

Some of the rules for which previous research exists include the following. Diabetes insipidus (13.20) has previously been identified as a potential cause of missed care and increased mortality (39, 40). Alkalosis (5.39–8.10) is associated with increased mortality (41). Alcohol dependence (2.34-11.69) can lead to various health problems of cognitive, cardiovascular, and gastrointestinal nature, among others (42). Each of these, in turn, can contribute to increased mortality and emergencies that lead to increased in-hospital mortality.

### 4.1. Limitations and Outlook

The present study has major limitations. First, only categorical data that changes slowly is considered. This excludes other interesting data like vital parameters and many biomarkers. Due to the rule-based approach, quantitative data have to be grouped into bins. Quickly changing data could easily be added to the method, but this would require analysis on a higher temporal resolution. Timestamps for all of the variables in the underlying dataset would help get more information from each case.

A second limitation is the lack of causal explanations. All the rules in both models are correlations between the variable and in-hospital mortality. They do not explain why this correlation exists. Future work is needed to introduce causal inference mechanisms into the presented approach.

There are several further possibilities for future work.

While the inconsistencies encountered in the dataset do not harm the proposed method's predictive performance, it might be helpful to remove them. However, it requires efforts to fix potential inconsistencies in thousands of clinical variables. Future research is needed to assess the consequences of resolving the inconsistencies as well as the potential for automated solutions to do so.

Longer rules, i.e., rules with more than one item on the left-hand side, can be created using ORs. This has not been analyzed in this study for two reasons. First, the predictive qualities are very good as-is, so more complex rules are not expected to bring much improvement. Second, more complex rules hinder interpretability. In the present form, the rules separate all variables and make them analyzable in isolation. This sets the proposed approach apart from Decision Trees, where many variables are merged into more complex rules, lowering the method's interpretability.

The proposed method was tested and validated using in-hospital mortality as an example. Other variables in MIMIC-IV could be used as the outcome of interest without changes to the model. This, of course, results in other sets of rules and different predictive performance, but the interpretable nature of the model remains the same.

One open question is the usability of the proposed method in clinics and hospitals. A usability study could be used to assess whether the proposed method is helpful to providers in realistic scenarios and whether it can replace or complement existing manual scoring methods. This is expected to highlight potential challenges in and benefits from the implementation of the proposed method.

## 5. Conclusion

We proposed a novel associative classification method to estimate a patient's in-hospital mortality risk. With a minimal model that uses patient-reported information that is quickly available and a full model that uses more information that is available at a later point in time, providers can objectively track a patient's in-hospital mortality risk. Apart from the high predictive performance, the rule-based nature of the proposed method allowed us to analyze which among around 180,000 variables play a role in the estimation of in-hospital mortality risk, resulting in a model with around 1,200 variables.

## Data Availability Statement

Publicly available datasets were analyzed in this study. This data can be found here: https://physionet.org/content/mimiciv/0.4/.

## Author Contributions

OH: concept, programming, analysis of the results, and writing of the manuscript. AM and ER: substantial revision of the work. All authors have approved the submitted version and take responsibility for the scientific integrity of the work.

## Funding

This project was funded by the Bavarian State Ministry of Science and the Arts, coordinated by the Bavarian Research Institute for Digital Transformation (bidt), and supported by the Bavarian Academic Forum (BayWISS)–Doctoral Consortium Health Research.

## Conflict of Interest

The authors declare that the research was conducted in the absence of any commercial or financial relationships that could be construed as a potential conflict of interest.

## Publisher's Note

All claims expressed in this article are solely those of the authors and do not necessarily represent those of their affiliated organizations, or those of the publisher, the editors and the reviewers. Any product that may be evaluated in this article, or claim that may be made by its manufacturer, is not guaranteed or endorsed by the publisher.

## Supplementary Material

Supplementary Table 1 The rules in the full model.

Supplementary Table 2 The rules in the minimal model.

Supplementary Table 3 One full Decision Tree model.
