# Supplementary material for: Rule-Based Models for Risk Estimation and Analysis of In-hospital Mortality in Emergency and Critical Care
Source: Front Med (Lausanne). 2021 Nov 8;8:785711. doi: 10.3389/fmed.2021.785711 (PMC8606583; doi:10.3389/fmed.2021.785711)
Supplement: Supplementary Table 3 — One full Decision Tree model. [file Table_3.DOCX]

|--- Prescription: 5% Dextrose = 0

| |--- Prescription: Morphine Sulfate = 0

| | |--- Diagnosis: Encounter for palliative care = 0

| | | |--- Procedure: Insertion of endotracheal tube = 0

| | | | |--- Diagnosis: Encounter for palliative care = 0

| | | | | |--- Prescription: Vasopressin = 0

| | | | | | |--- Diagnosis: Less than 24 completed weeks of gestation = 0

| | | | | | | |--- weights: [169939, 11562] class: 0

| | | | | | |--- Diagnosis: Less than 24 completed weeks of gestation = 1

| | | | | | | |--- weights: [1, 1061] class: 1

| | | | | |--- Prescription: Vasopressin = 1

| | | | | | |--- Prescription: Dexmedetomidine = 0

| | | | | | | |--- weights: [56, 1229] class: 1

| | | | | | |--- Prescription: Dexmedetomidine = 1

| | | | | | | |--- weights: [27, 0] class: 0

| | | | |--- Diagnosis: Encounter for palliative care = 1

| | | | | |--- Prescription: HYDROmorphone (Dilaudid) = 0

| | | | | | |--- Ward: Hematology/Oncology = 0

| | | | | | | |--- weights: [83, 363] class: 1

| | | | | | |--- Ward: Hematology/Oncology = 1

| | | | | | | |--- weights: [44, 0] class: 0

| | | | | |--- Prescription: HYDROmorphone (Dilaudid) = 1

| | | | | | |--- Prescription: Ibuprofen = 0

| | | | | | | |--- weights: [61, 1201] class: 1

| | | | | | |--- Prescription: Ibuprofen = 1

| | | | | | | |--- weights: [7, 0] class: 0

| | | |--- Procedure: Insertion of endotracheal tube = 1

| | | | |--- Prescription: Hepatitis B Vaccine = 0

| | | | | |--- Procedure: Cardiopulmonary resuscitation, not otherwise specified = 0

| | | | | | |--- Prescription: Norepinephrine = 0

| | | | | | | |--- weights: [568, 1536] class: 1

| | | | | | |--- Prescription: Norepinephrine = 1

| | | | | | | |--- weights: [31, 922] class: 1

| | | | | |--- Procedure: Cardiopulmonary resuscitation, not otherwise specified = 1

| | | | | | |--- Prescription: Potassium Chl 20 mEq / 1000 mL D5 1/2 NS = 0

| | | | | | | |--- weights: [6, 1340] class: 1

| | | | | | |--- Prescription: Potassium Chl 20 mEq / 1000 mL D5 1/2 NS = 1

| | | | | | | |--- weights: [3, 0] class: 0

| | | | |--- Prescription: Hepatitis B Vaccine = 1

| | | | | |--- Prescription: NEO*PO*Spironolactone = 0

| | | | | | |--- Diagnosis: Transitory tachypnea of newborn = 0

| | | | | | | |--- weights: [208, 0] class: 0

| | | | | | |--- Diagnosis: Transitory tachypnea of newborn = 1

| | | | | | | |--- weights: [9, 0] class: 0

| | | | | |--- Prescription: NEO*PO*Spironolactone = 1

| | | | | | |--- weights: [0, 28] class: 1

| | |--- Diagnosis: Encounter for palliative care = 1

| | | |--- Prescription: Glycopyrrolate = 0

| | | | |--- Prescription: Polyethylene Glycol = 0

| | | | | |--- Prescription: Spironolactone = 0

| | | | | | |--- Diagnosis: Adverse effect of antineoplastic and immunosuppressive drugs, initial encounter = 0

| | | | | | | |--- weights: [171, 2458] class: 1

| | | | | | |--- Diagnosis: Adverse effect of antineoplastic and immunosuppressive drugs, initial encounter = 1

| | | | | | | |--- weights: [12, 0] class: 0

| | | | | |--- Prescription: Spironolactone = 1

| | | | | | |--- weights: [18, 0] class: 0

| | | | |--- Prescription: Polyethylene Glycol = 1

| | | | | |--- Ward: Medicine = 0

| | | | | | |--- Prescription: Calcium Carbonate = 0

| | | | | | | |--- weights: [113, 503] class: 1

| | | | | | |--- Prescription: Calcium Carbonate = 1

| | | | | | | |--- weights: [23, 0] class: 0

| | | | | |--- Ward: Medicine = 1

| | | | | | |--- Diagnosis: Aphasia = 0

| | | | | | | |--- weights: [83, 0] class: 0

| | | | | | |--- Diagnosis: Aphasia = 1

| | | | | | | |--- weights: [0, 28] class: 1

| | | |--- Prescription: Glycopyrrolate = 1

| | | | |--- Diagnosis: Aphasia = 0

| | | | | |--- Prescription: Finasteride = 0

| | | | | | |--- Diagnosis: Diaphragmatic hernia without obstruction or gangrene = 0

| | | | | | | |--- weights: [20, 3798] class: 1

| | | | | | |--- Diagnosis: Diaphragmatic hernia without obstruction or gangrene = 1

| | | | | | | |--- weights: [2, 0] class: 0

| | | | | |--- Prescription: Finasteride = 1

| | | | | | |--- weights: [2, 0] class: 0

| | | | |--- Diagnosis: Aphasia = 1

| | | | | |--- weights: [3, 0] class: 0

| |--- Prescription: Morphine Sulfate = 1

| | |--- Prescription: Scopolamine Patch = 0

| | | |--- Prescription: D5W = 0

| | | | |--- Diagnosis: Do not resuscitate = 0

| | | | | |--- Diagnosis: Do not resuscitate status = 0

| | | | | | |--- Prescription: Glycopyrrolate = 0

| | | | | | | |--- weights: [20100, 2513] class: 0

| | | | | | |--- Prescription: Glycopyrrolate = 1

| | | | | | | |--- weights: [28, 670] class: 1

| | | | | |--- Diagnosis: Do not resuscitate status = 1

| | | | | | |--- Ward: Med/Surg/Trauma = 0

| | | | | | | |--- weights: [303, 3519] class: 1

| | | | | | |--- Ward: Med/Surg/Trauma = 1

| | | | | | | |--- weights: [40, 28] class: 0

| | | | |--- Diagnosis: Do not resuscitate = 1

| | | | | |--- Service: ORTHO = 0

| | | | | | |--- Diagnosis: Encounter for palliative care = 0

| | | | | | | |--- weights: [160, 1033] class: 1

| | | | | | |--- Diagnosis: Encounter for palliative care = 1

| | | | | | | |--- weights: [121, 4692] class: 1

| | | | | |--- Service: ORTHO = 1

| | | | | | |--- Prescription: CefazoLIN = 0

| | | | | | | |--- weights: [44, 0] class: 0

| | | | | | |--- Prescription: CefazoLIN = 1

| | | | | | | |--- weights: [3, 0] class: 0

| | | |--- Prescription: D5W = 1

| | | | |--- Prescription: Oxycodone-Acetaminophen = 0

| | | | | |--- Prescription: Nitroglycerin = 0

| | | | | | |--- Prescription: Potassium Chl 20 mEq / 1000 mL D5 1/2 NS = 0

| | | | | | | |--- weights: [668, 7987] class: 1

| | | | | | |--- Prescription: Potassium Chl 20 mEq / 1000 mL D5 1/2 NS = 1

| | | | | | | |--- weights: [175, 307] class: 1

| | | | | |--- Prescription: Nitroglycerin = 1

| | | | | | |--- Prescription: Vancomycin = 0

| | | | | | | |--- weights: [252, 195] class: 0

| | | | | | |--- Prescription: Vancomycin = 1

| | | | | | | |--- weights: [55, 363] class: 1

| | | | |--- Prescription: Oxycodone-Acetaminophen = 1

| | | | | |--- Prescription: Norepinephrine = 0

| | | | | | |--- Ward: Medical Intensive Care Unit (MICU) = 0

| | | | | | | |--- weights: [551, 279] class: 0

| | | | | | |--- Ward: Medical Intensive Care Unit (MICU) = 1

| | | | | | | |--- weights: [17, 140] class: 1

| | | | | |--- Prescription: Norepinephrine = 1

| | | | | | |--- Prescription: Dexmedetomidine = 0

| | | | | | | |--- weights: [32, 391] class: 1

| | | | | | |--- Prescription: Dexmedetomidine = 1

| | | | | | | |--- weights: [11, 0] class: 0

| | |--- Prescription: Scopolamine Patch = 1

| | | |--- Service: ORTHO = 0

| | | | |--- Service: PSURG = 0

| | | | | |--- Service: SURG = 0

| | | | | | |--- Prescription: Pregabalin = 0

| | | | | | | |--- weights: [244, 12707] class: 1

| | | | | | |--- Prescription: Pregabalin = 1

| | | | | | | |--- weights: [15, 0] class: 0

| | | | | |--- Service: SURG = 1

| | | | | | |--- Gender: M = 0

| | | | | | | |--- weights: [68, 28] class: 0

| | | | | | |--- Gender: M = 1

| | | | | | | |--- weights: [13, 168] class: 1

| | | | |--- Service: PSURG = 1

| | | | | |--- weights: [56, 0] class: 0

| | | |--- Service: ORTHO = 1

| | | | |--- Diagnosis: Displaced intertrochanteric fracture of left femur, initial encounter for closed fracture = 0

| | | | | |--- weights: [84, 0] class: 0

| | | | |--- Diagnosis: Displaced intertrochanteric fracture of left femur, initial encounter for closed fracture = 1

| | | | | |--- weights: [0, 28] class: 1

|--- Prescription: 5% Dextrose = 1

| |--- Prescription: Fentanyl Citrate = 0

| | |--- Prescription: Morphine Sulfate = 0

| | | |--- Prescription: NORepinephrine = 0

| | | | |--- Diagnosis: Encounter for palliative care = 0

| | | | | |--- Diagnosis: Do not resuscitate status = 0

| | | | | | |--- Procedure: Insertion of endotracheal tube = 0

| | | | | | | |--- weights: [23767, 5334] class: 0

| | | | | | |--- Procedure: Insertion of endotracheal tube = 1

| | | | | | | |--- weights: [185, 1480] class: 1

| | | | | |--- Diagnosis: Do not resuscitate status = 1

| | | | | | |--- Prescription: HYDROmorphone (Dilaudid) = 0

| | | | | | | |--- weights: [678, 1145] class: 1

| | | | | | |--- Prescription: HYDROmorphone (Dilaudid) = 1

| | | | | | | |--- weights: [119, 1927] class: 1

| | | | |--- Diagnosis: Encounter for palliative care = 1

| | | | | |--- Prescription: HYDROmorphone (Dilaudid) = 0

| | | | | | |--- Service: CMED = 0

| | | | | | | |--- weights: [79, 391] class: 1

| | | | | | |--- Service: CMED = 1

| | | | | | | |--- weights: [21, 0] class: 0

| | | | | |--- Prescription: HYDROmorphone (Dilaudid) = 1

| | | | | | |--- Diagnosis: Secondary malignant neoplasm of small intestine = 0

| | | | | | | |--- weights: [84, 2737] class: 1

| | | | | | |--- Diagnosis: Secondary malignant neoplasm of small intestine = 1

| | | | | | | |--- weights: [6, 0] class: 0

| | | |--- Prescription: NORepinephrine = 1

| | | | |--- Prescription: Vasopressin = 0

| | | | | |--- Prescription: Enoxaparin Sodium = 0

| | | | | | |--- Ward: Medicine = 0

| | | | | | | |--- weights: [278, 3351] class: 1

| | | | | | |--- Ward: Medicine = 1

| | | | | | | |--- weights: [192, 503] class: 1

| | | | | |--- Prescription: Enoxaparin Sodium = 1

| | | | | | |--- Diagnosis: Encounter for palliative care = 0

| | | | | | | |--- weights: [63, 0] class: 0

| | | | | | |--- Diagnosis: Encounter for palliative care = 1

| | | | | | | |--- weights: [0, 28] class: 1

| | | | |--- Prescription: Vasopressin = 1

| | | | | |--- Prescription: Zinc Sulfate = 0

| | | | | | |--- Diagnosis: Sepsis due to Escherichia coli [E. coli] = 0

| | | | | | | |--- weights: [58, 3658] class: 1

| | | | | | |--- Diagnosis: Sepsis due to Escherichia coli [E. coli] = 1

| | | | | | | |--- weights: [5, 0] class: 0

| | | | | |--- Prescription: Zinc Sulfate = 1

| | | | | | |--- weights: [5, 0] class: 0

| | |--- Prescription: Morphine Sulfate = 1

| | | |--- Ward: Cardiac Surgery = 0

| | | | |--- Service: SURG = 0

| | | | | |--- Prescription: Scopolamine Patch = 0

| | | | | | |--- Service: ORTHO = 0

| | | | | | | |--- weights: [4996, 30998] class: 1

| | | | | | |--- Service: ORTHO = 1

| | | | | | | |--- weights: [784, 391] class: 0

| | | | | |--- Prescription: Scopolamine Patch = 1

| | | | | | |--- Service: PSURG = 0

| | | | | | | |--- weights: [137, 21140] class: 1

| | | | | | |--- Service: PSURG = 1

| | | | | | | |--- weights: [34, 0] class: 0

| | | | |--- Service: SURG = 1

| | | | | |--- Prescription: Vancomycin = 0

| | | | | | |--- Diagnosis: Encounter for palliative care = 0

| | | | | | | |--- weights: [1582, 475] class: 0

| | | | | | |--- Diagnosis: Encounter for palliative care = 1

| | | | | | | |--- weights: [2, 195] class: 1

| | | | | |--- Prescription: Vancomycin = 1

| | | | | | |--- Ethnicity: BLACK/AFRICAN AMERICAN = 0

| | | | | | | |--- weights: [390, 1340] class: 1

| | | | | | |--- Ethnicity: BLACK/AFRICAN AMERICAN = 1

| | | | | | | |--- weights: [50, 0] class: 0

| | | |--- Ward: Cardiac Surgery = 1

| | | | |--- Prescription: Nitroglycerin = 0

| | | | | |--- Prescription: Ipratropium Bromide Neb = 0

| | | | | | |--- Ward: Coronary Care Unit (CCU) = 0

| | | | | | | |--- weights: [73, 0] class: 0

| | | | | | |--- Ward: Coronary Care Unit (CCU) = 1

| | | | | | | |--- weights: [8, 84] class: 1

| | | | | |--- Prescription: Ipratropium Bromide Neb = 1

| | | | | | |--- Insurance: Other = 0

| | | | | | | |--- weights: [7, 279] class: 1

| | | | | | |--- Insurance: Other = 1

| | | | | | | |--- weights: [7, 0] class: 0

| | | | |--- Prescription: Nitroglycerin = 1

| | | | | |--- Procedure: Insertion of endotracheal tube = 0

| | | | | | |--- weights: [1845, 0] class: 0

| | | | | |--- Procedure: Insertion of endotracheal tube = 1

| | | | | | |--- Ward: PACU = 0

| | | | | | | |--- weights: [16, 0] class: 0

| | | | | | |--- Ward: PACU = 1

| | | | | | | |--- weights: [10, 84] class: 1

| |--- Prescription: Fentanyl Citrate = 1

| | |--- Prescription: Insulin Glargine (CVICU Protocol) = 0

| | | |--- Prescription: Vasopressin = 0

| | | | |--- Prescription: Morphine Sulfate = 0

| | | | | |--- Prescription: NORepinephrine = 0

| | | | | | |--- Diagnosis: Encounter for palliative care = 0

| | | | | | | |--- weights: [2062, 4161] class: 1

| | | | | | |--- Diagnosis: Encounter for palliative care = 1

| | | | | | | |--- weights: [17, 1061] class: 1

| | | | | |--- Prescription: NORepinephrine = 1

| | | | | | |--- Prescription: Lisinopril = 0

| | | | | | | |--- weights: [671, 8322] class: 1

| | | | | | |--- Prescription: Lisinopril = 1

| | | | | | | |--- weights: [175, 223] class: 1

| | | | |--- Prescription: Morphine Sulfate = 1

| | | | | |--- Prescription: Oxycodone-Acetaminophen = 0

| | | | | | |--- Procedure: Open and other replacement of aortic valve with tissue graft = 0

| | | | | | | |--- weights: [1251, 35941] class: 1

| | | | | | |--- Procedure: Open and other replacement of aortic valve with tissue graft = 1

| | | | | | | |--- weights: [26, 0] class: 0

| | | | | |--- Prescription: Oxycodone-Acetaminophen = 1

| | | | | | |--- Prescription: Potassium Chl 20 mEq / 1000 mL D5 1/2 NS = 0

| | | | | | | |--- weights: [146, 419] class: 1

| | | | | | |--- Prescription: Potassium Chl 20 mEq / 1000 mL D5 1/2 NS = 1

| | | | | | | |--- weights: [35, 0] class: 0

| | | |--- Prescription: Vasopressin = 1

| | | | |--- Prescription: Warfarin = 0

| | | | | |--- Prescription: Clonidine Patch 0.2 mg/24 hr = 0

| | | | | | |--- Diagnosis: Thoracoscopic Lobectomy Of Lung = 0

| | | | | | | |--- weights: [438, 44208] class: 1

| | | | | | |--- Diagnosis: Thoracoscopic Lobectomy Of Lung = 1

| | | | | | | |--- weights: [4, 0] class: 0

| | | | | |--- Prescription: Clonidine Patch 0.2 mg/24 hr = 1

| | | | | | |--- weights: [6, 0] class: 0

| | | | |--- Prescription: Warfarin = 1

| | | | | |--- Prescription: Tacrolimus = 0

| | | | | | |--- Prescription: Pantoprazole (Granules for DR Suspension) = 0

| | | | | | | |--- weights: [173, 3491] class: 1

| | | | | | |--- Prescription: Pantoprazole (Granules for DR Suspension) = 1

| | | | | | | |--- weights: [9, 0] class: 0

| | | | | |--- Prescription: Tacrolimus = 1

| | | | | | |--- weights: [10, 0] class: 0

| | |--- Prescription: Insulin Glargine (CVICU Protocol) = 1

| | | |--- Prescription: Sodium Bicarbonate = 0

| | | | |--- Diagnosis: Encounter for palliative care = 0

| | | | | |--- Prescription: D10W = 0

| | | | | | |--- Prescription: Furosemide = 0

| | | | | | | |--- weights: [26, 56] class: 1

| | | | | | |--- Prescription: Furosemide = 1

| | | | | | | |--- weights: [923, 0] class: 0

| | | | | |--- Prescription: D10W = 1

| | | | | | |--- Diagnosis: Other surgical procedures as the cause of abnormal reaction of the patient, or of later complication, without mention of misadventure at the time of the procedure = 0

| | | | | | | |--- weights: [4, 0] class: 0

| | | | | | |--- Diagnosis: Other surgical procedures as the cause of abnormal reaction of the patient, or of later complication, without mention of misadventure at the time of the procedure = 1

| | | | | | | |--- weights: [0, 56] class: 1

| | | | |--- Diagnosis: Encounter for palliative care = 1

| | | | | |--- Prescription: TraMADol = 0

| | | | | | |--- weights: [0, 112] class: 1

| | | | | |--- Prescription: TraMADol = 1

| | | | | | |--- weights: [1, 0] class: 0

| | | |--- Prescription: Sodium Bicarbonate = 1

| | | | |--- Prescription: Vancomycin = 0

| | | | | |--- weights: [8, 0] class: 0

| | | | |--- Prescription: Vancomycin = 1

| | | | | |--- Prescription: LOPERamide = 0

| | | | | | |--- Diagnosis: Major depressive disorder, single episode, unspecified = 0

| | | | | | | |--- weights: [35, 1368] class: 1

| | | | | | |--- Diagnosis: Major depressive disorder, single episode, unspecified = 1

| | | | | | | |--- weights: [5, 0] class: 0

| | | | | |--- Prescription: LOPERamide = 1

| | | | | | |--- weights: [5, 0] class: 0
